# Experimental Study on Dust Suppression Effect and Performance of New Nano-Composite Dust Suppressant

**DOI:** 10.3390/ijerph19106288

**Published:** 2022-05-22

**Authors:** Ming Li, Xinzhu Song, Gang Li, Jiao Tang, Zhi Li

**Affiliations:** 1 School of Resources and Safety Engineering, Central South University, Changsha 410083, China; liming_csu@csu.edu.cn (M.L.); tang_jiao2022@163.com (J.T.); lizhi89@csu.edu.cn (Z.L.); 2 Sinosteel Maanshan General Institute of Mining Research Co., Ltd., Maanshan 243000, China; hunankedaligang@163.com

**Keywords:** dust suppressant, nanometer material, nanofluid, moisturizing performance, wettability

## Abstract

In this paper, a nano-composite dust suppressant has been proposed to make up for the deficiency in wettability and moisturizing performance of a nanofluid dust suppressant. The nanometer material *Al*_2_*O*_3_, super absorbent polymer, carboxyl methyl starch sodium, and polyacrylamide were selected as effective components of it. The surface tension of the solution, evaporation resistance, and uniaxial compressive strength (UCS) were chosen as evaluation index to compare the suppression performance, these dust suppressants include the water, nanofluid dust suppressant and nano-composite dust suppressant, and the surface morphology of each tested material was observed by micro image analysis system. It was found that the surface tension and water loss rates of the nano-composite dust suppressants, respectively, decreased by 31.96% and 7.1%, and the maximum UCS increased by 31.82% compared with data of nanofluid dust suppressants. Since the nano-composite dust suppressant has good dispersion, permeability and bond performance, the suppressant film has fewer micro-cracks from the photos of microscopic image; it can improve the compactness and integrity of dust consolidation to prevent the evaporation of water and dust re-entrainment.

## 1. Introduction

**Figure 1:**
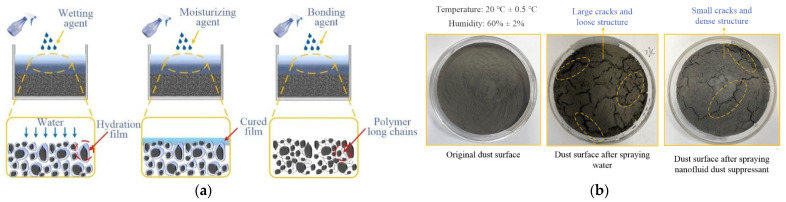
The mechanism of dust suppression and the experimental photos of dust surface. (**a**) The mechanism of dust suppression. (**b**) The experimental photos of dust surface.

Dust pollution is one of many environmental problems faced by mining production. As a new dust control technology, chemical dust suppressant has been widely studied and applied in recent years. The mechanism of chemical dust suppressant can be divided into wetting, moisturizing, bonding, and composite effects, as shown in Figure 1a. Many researchers have developed chemical dust suppressant with different functions. Wang et al. experimentally studied the wettability between dust suppressants and different coal dust [1]. Zhou et al. evaluated the comprehensive wettability of dust suppressant on lignite dust [2]. Zhang et al. developed and tested a bonding dust suppressant suitable for open-pit coal mine [3]. Liu et al. studied the water retention performance of NCZ composite dust suppressant and its wettability on hydrophobic coal dust [4]. Cheng et al. developed a composite dust suppressant for coal dust wetting [5]. Jin et al. prepared and characterized a composite dust suppressant for coal mine [6]. Generally speaking, the components of wetting type dust suppressant are mostly surfactants, such as water-absorbent resin dust suppressant with cation [7] and anions Cl-, Br- [8], non-ionic surfactants APG0810 and PPG400 [9], 2-Alkyl-1-diethylenediaminimidazoline gemini surfactants [10]. Another type of wetting suppressant is hygroscopic agent, such as carboxymethyl chitosan [11], which can absorb water in the environment to keep the dust moistness. The components of moisturizing type are mostly inorganic salts [12], super absorbent polymer [13], some of them can form a cured film on the dust surface after absorbing water, and the film can prevent water evaporation. The components of bonding type are mostly high polymeric organics, such as SAPs [14], acrylic acid and acrylamide polymer [15], which can effectively consolidate dust particles through polymer long chains. 

The nano materials have obvious size effect, high surface energy and strong adsorption performance, and they are often used to prepare nanofluids as adsorbents [16]. According to the functions of dust suppression, Bai et al. [17] comparative studied the suppressant performance of several nano materials, she found that nanoparticles can penetrate into the micro-gap between dust and fill the gap to enhance the consistency of dust surface. Since the nanoparticles have strong adsorption performance, it can adhere and agglomerate the surrounding dust particles and effectively reduce the generation of large cracks, as shown in Figure 1b. 

However, the deficiency of suppressant performance is wettability and moisturizing performance for the nanofluid dust suppressants, and its dust suppression effect is relatively single. Considering the shortcomings of the nanofluid dust suppressants and the advantages of the traditional dust suppressant, the paper puts forward the ideal of the nano-composite dust suppressant, its component includes traditional dust suppressants and nanofluid materials.

## 2. Materials and Methods

### 2.1. Materials

The functions of nano-composite dust suppressant combined with the wetting, moisturizing and bonding properties, its component are 30 nm $Al_{2}O_{3}$ nanofluid (Produced by Zhongmai metal materials Co., Ltd., Lianyungang, China), super absorbent polymer (SAP, produced by Hebei Yanxing Chemical Co., Ltd., Cangzhou, China), carboxyl methyl starch sodium (CMS, produced by Hebei Yanxing Chemical Co., Ltd., Cangzhou, China) and polyacrylamide (PAM, produced by Sinopharm Chemical Reagent Co., Ltd., Shanghai, China). 

**Figure 2:**
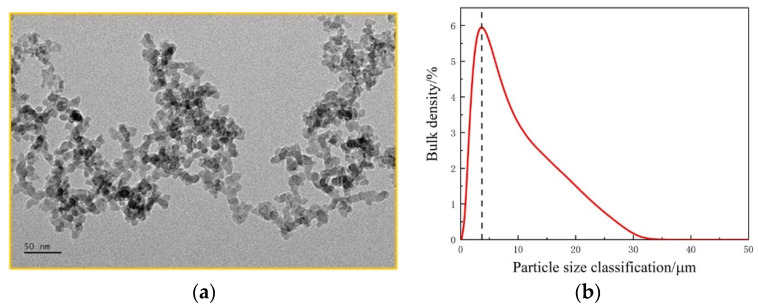
Analysis of experimental materials. (**a**) The photo of TEM. (**b**) Particle size distribution of experimental dust sample.

The nanometer material $Al_{2}O_{3}$ and experimental dust sample were tested by the TEM and laser granularity analyzer, respectively, as shown in Figure 2.

The nanoparticles have certain dispersibility and the particle size meets the requirements of experimental research from Figure 2a. The particle size of testing dust samples is shown in Figure 2b.

### 2.2. Methods

#### 2.2.1. Preparation Method of Experimental Materials

**Figure 3:**
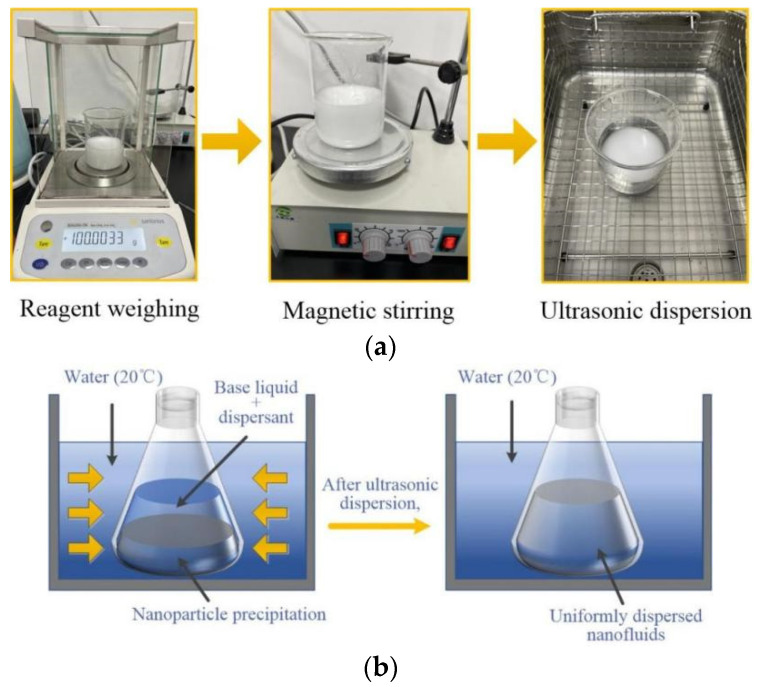
Preparation method of nanofluid dust suppressant. (**a**) Preparation process of nanofluid dust suppressant. (**b**) State of nanofluid dust suppressant before and after ultrasonic dispersion.

The experiment adopts a “two-step method” [18] to prepare nanofluid dust suppressant, the dispersant is SDBS solution. The agglomerated nanoparticles can be broken by magnetic stirring and ultrasonic dispersion (Figure 3a), the dispersant SDBS can penetrate into the nanoparticles and quickly coated them, and prevent the nanoparticles from contacting and agglomerating with each other. The nanofluid dust suppressant can maintain stable dispersion state, as shown in Figure 3b.

In order to obtain the optimized formulation of nano-composite dust suppressant, three different concentrations of each component were selected according to the single-factor pre-experiment, and nine groups of nano-composite dust suppressants were prepared by four factor and three-level orthogonal test, as shown in Table 1.

#### 2.2.2. Test Method of Dust Suppression Performance

**Figure 4:**
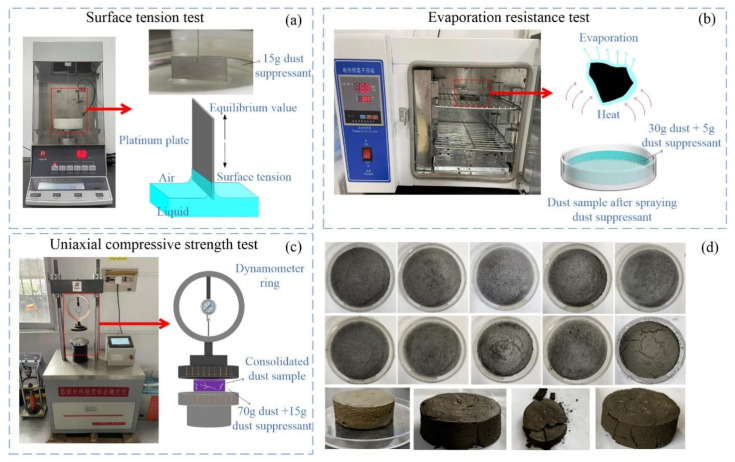
Test method of nano-composite dust suppressant performance. (**a**) Surface tension test. (**b**) Evaporation resistance test. (**c**) Uniaxial compressive strength test. (**d**) Experimental pictures.

The surface tension value, evaporation resistance performance [19] and the UCS [20] of dust pile were selected to evaluate the performance of nano-composite dust suppressants with different ratios, the test method and some experimental pictures are shown in Figure 4.

The surface tension of dust suppressant solution is measured three times by surface tension meter, and the average value is the final result, as shown in Figure 4a.

The test method of evaporation resistance performance as shown in Figure 4b, the dried dust sample (30 g) is evenly sprinkle into the culture plate with a diameter of 60 mm, and the nano-composite dust suppressant (5 g) is evenly spray on dust surface, then the mass of test sample is weighed as the $W_{0}$, the test sample is put into the drying oven at 60 °C and weighed every hour, the mass of test sample is weighed as the $W_{i}$, and the water loss rate can be calculated by Equation (1).

(1) $$\alpha =\frac{W_{0}-W_{i}}{W_{l}}\times 100\%$$

In Equation (1), α (%) is the water loss rate; $W_{i}$ (g) is the mass of the *i*-th test weight; $W_{0}$ (g) is the initial mass of test sample, including the mass of dust and dust suppressant; $W_{l}$ (g) is the mass of spraying dust suppressant.

The test method of UCS of dust pile as shown in Figure 4c, the dust sample (70 g) is put into cylindrical rubber mold with diameter of 5 cm and height of 2 cm, the nano-composite dust suppressant (15 g) is spray evenly on dust surface, the test sample is dried until the mass is basically unchanged. After that, the testing dust sample is taken out the mold and the value of the UCS is measured by using comprehensive strength tester.

## 3. Results and Discussion

The data of surface tension of solution, evaporation resistance performance and the UCS were obtained by the experiment, the surfaces of dust sample were tested by micro image analysis system to get the morphology.

### 3.1. Surface Tension Analysis

**Figure 5:**
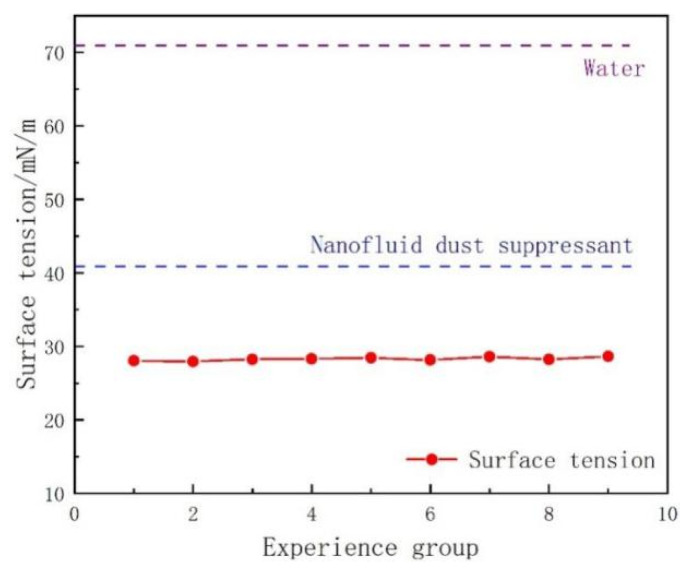
Surface tension value of dust suppressant.

During the preparation of the nanofluid, the surfactant SDBS was added as the dispersant, which can reduce the surface tension of dust suppressant. The results of the surface tension of each group in the experiment are shown in Figure 5.

It can be seen that the surface tension value of the $Al_{2}O_{3}$ nanofluid dust suppressant is 42.26% lower than that of water, and the average value of nano-composite dust suppressant is 31.96% lower than that of $Al_{2}O_{3}$ nanofluid dust suppressant from Figure 5. The value of nano-composite dust suppressant is 27~29 mN/m, while it is 30~40 mN/m for traditional chemical dust suppressant, it can be proved that the nanofluid and traditional chemical composition have good synergistic effect, which provides the possibility to further improve the wettability.

### 3.2. Evaporation Resistance Performance Analysis

**Figure 6:**
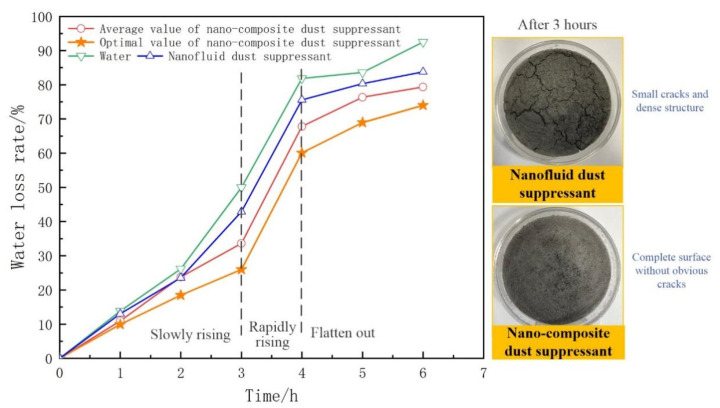
The results of water loss rate.

The average and optimal value of water loss rate are discussed, and the results as shown in Figure 6.

The water loss rate of nano-composite dust suppressant rises relatively slowly when it was dried for 3 h, it can be proved that the nano-composite dust suppressant has a good evaporation resistance performance from Figure 6. The photos show the surfaces sprayed with nanofluid dust suppressant and nano-composite dust suppressant when they were dried for 3 h. The dust surface sprayed with nanofluid was drier and showed big cracks in the surface sprayed with the nano-composite (which remained wet and smooth). This is due to the fact that SAP can adsorb moisture and form a gel structure to fix water, CMS can form a cured film on the dust surface, which can lock the moisture inside the dust pile. 

When these test dust samples were dried for 3~4 h, all of the water loss rates increased rapidly, and the continuous drying destroyed the film integrity of the dust suppressant. After drying for 4 h, the change trend of water loss rate is gentle, most of the water has evaporated when it was not prevented by the cured film, and the value of water close to 100%.

The value of the optimal group is lower than any others, the results show that the moisturizing performance of nanofluid dust suppressant can be significantly improved by compounding with traditional dust suppressant.

### 3.3. Consolidation Strength of Dust Pile Analysis

**Figure 7:**
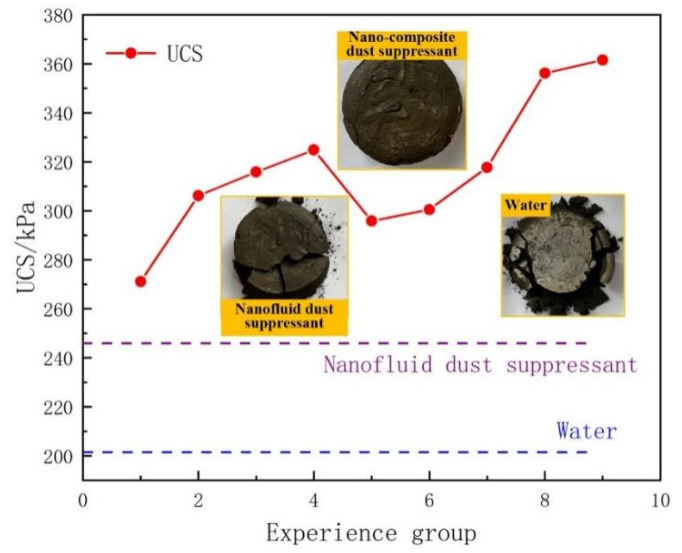
Test results of the UCS.

The consolidation effect of dust suppressant can improve the strength of the dust pile, the dust re-entrainment is not easy to appear under the external force. The test results of the maximum UCS are compared and analyzed, as shown in Figure 7.

The water test sample was broken into many small pieces and a large number of granular dust particles from photos in Figure 7. The nanofluid test sample was broken into several pieces and some granular dust particles, while there are some small cracks appeared in the test sample of nano-composite, and the dust pile was still intact.

It is proved that nanofluid dust suppressant can improve the compactness and strength of the test samples by infiltrating nano materials into the gap of the dust pile, but the adsorption of nano particles cannot withstand the action of large external force. 

The optimal value of the maximum UCS of nano-composite dust suppressant is 31.82% higher than that of nanofluid dust suppressant and 44.18% higher than that of water from the experimental data.

### 3.4. Dust Surface Morphology and Causes Analysis

**Figure 8:**
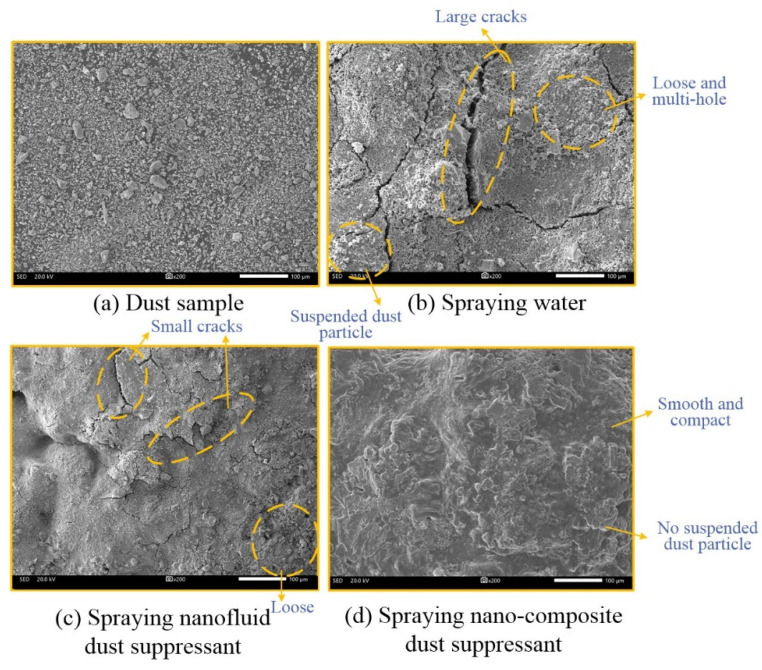
Photos of SEM.

The dust surface of test sample was analyzed by SEM (SED = 20 kV, WD = 8.2 mm), and the results are shown in Figure 8.

There are some obvious cracks on the surface of water test sample from Figure 8b, many fine and angular dust particles was distributed on the surface, and the structure of the dust pile was relatively loose. 

The opening size of cracks was obviously decreased for nanofluid dust suppressant, while the dust surface was still rough and there are many fine holes in the surface from the Figure 8c.

It can not be seen some small crack and loose dust particle in the surface of nano-composite dust suppressant, the surface structure is relatively continuous and the consolidation layer was relatively intact and uniform from Figure 8d. 

**Figure 9:**
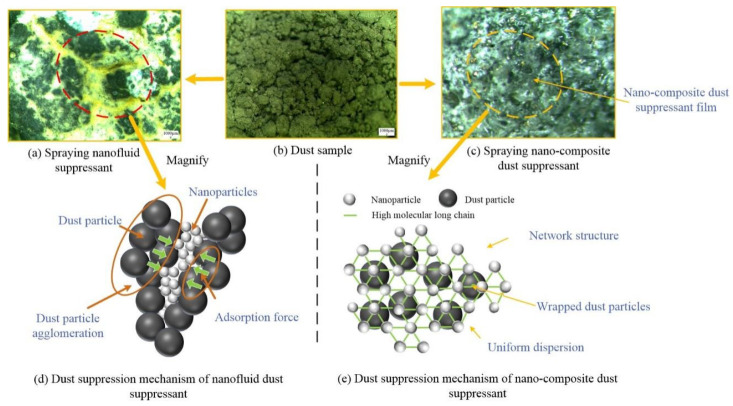
Suppression mechanism of nano-composite dust suppressant.

In order to compare the difference of dust surface morphology and discuss the dust suppression mechanism, Figure 9 is made according to the experimental pictures.

In Figure 9a, the nanofluid suppressant penetrates into the gap, and agglomerates the dust particles by their adsorption force, as shown in Figure 9d, but it can be seen that the surface is still exposed to the air and nanoparticles distributed unevenly on the dust surface, it can not prevent the evaporation of water and dust re-entrainment. The consolidation strength can be decreased due to the gap. When the suppressant layer is broken, dust will directly diffuse into the air again. 

In Figure 9c, the nano-composite dust suppressant forms a film on the dust surface. This film is a network structure composed of nanoparticles and polymer long chains of PAM, as shown in Figure 9e, it can effectively adsorb and tightly wrap the dust particles, and improve the consolidation strength of dust to reduce the generation of cracks. In addition, the dense film can prevent the evaporation of water and dust re-entrainment, when a lot of water exist in the dust pile, the dust particles will adhere to each other due to the capillary force of water. 

The nano-composite dust suppressant can adsorb and bond the surrounding dust particles and improve the overall integrity and strength of the suppressant film. The traditional components of nano-composite dust suppressant improve the moisturizing performance and consolidation ability compared with nanofluid dust suppressant. The comprehensive dust suppression effect of nano-composite dust suppressant has been significantly improved.

## 4. Conclusions

The performance of dust suppression has been studied among the nanofluid dust suppressant, nano-composite dust suppressant and water, and the difference of the mechanism of dust suppression was discussed.

(1) A new formulation of nano-composite dust suppressant was proposed. The nanofluid dust suppressant has high-efficiency adsorption performance, which can effectively agglomerate surrounding dust particles, but it is poor in wetting and moisturizing functions from the mechanism of dust suppression. The traditional components of dust suppressant were proposed to improve the comprehensive performance of nanofluid dust suppressant. The nanometer material $Al_{2}O_{3}$, super absorbent polymer, carboxyl methyl starch sodium, and polyacrylamide were selected as effective components of the nano-composite dust suppressant. The experimental results show that the surface tension of solution and water loss rates of nano-composite dust suppressants, respectively, decreased by 31.96% and 7.1%, the maximum UCS increased by 31.82% compared with data of nanofluid dust suppressant.
(2) The nano-composite dust suppressant has multiple dust suppression effects. The nano-composite dust suppressant has good dispersion, permeability, and bond performance, and the suppressant film has fewer micro-cracks from the photos of microscopic image. The nanoparticles can adsorb and bond the surrounding dust particles and improve the overall integrity and strength of the suppressant film, the traditional components can improve the moisturizing performance and consolidation ability compared with nanofluid dust suppressant, and the dense film can prevent the evaporation of water and dust re-entrainment. The comprehensive dust suppression effect of nano-composite dust suppressant has been significantly improved.

## Figures and Tables

**Table 1:** Orthogonal experiment table.

Group No.	Factors			
	Nanofluid/g	SAP/g	CMS/g	PAM/g
1	1.0	0.2	0.5	0.002
2	1.0	0.3	1.0	0.004
3	1.0	0.4	1.5	0.006
4	1.5	0.2	1.0	0.006
5	1.5	0.3	1.5	0.002
6	1.5	0.4	0.5	0.004
7	2.0	0.2	1.5	0.004
8	2.0	0.3	0.5	0.006
9	2.0	0.4	1.0	0.002

## Data Availability

The data presented in this study are available in the Web of Science core database.
